# Thin-Film Stabilization and Magnetism of η‑Carbide-Type Iron Nitrides

**DOI:** 10.1021/acs.chemmater.6c00901

**Published:** 2026-06-17

**Authors:** Baptiste Julien, Abrar Rauf, Liam A. V. Nagle-Cocco, Rebecca W. Smaha, Wenhao Sun, Andriy Zakutayev, Sage R. Bauers

**Affiliations:** † Materials Science Center, 53405 National Laboratory of the Rockies, Golden, Colorado 80401, United States; ‡ Department of Materials Science and Engineering, 1259 University of Michigan, Ann Arbor, Michigan 48109-1079, United States; § Stanford Synchrotron Radiation Lightsource, Stanford University, Menlo Park, California 94025, United States

## Abstract

Transition-metal nitrides in η-carbide-type structures exhibit unusual bonding motifs and proximity to magnetic instabilities. Yet they remain unexplored in thin-film form due to the difficulty of stabilizing nitrogen-poor ternaries among competing phases. Here, we report the thin-film synthesis and phase-stability mapping of the η-nitride systems Fe–W–N and Fe–Mo–N. Amorphous Fe–M–N (M = W, Mo) combinatorial libraries deposited by reactive cosputtering crystallize upon rapid thermal annealing, enabling systematic identification of synthesis windows as a function of composition and annealing temperature. Using laboratory powder X-ray diffraction and synchrotron grazing incidence wide-angle X-ray scattering, we establish that Fe_3_Mo_3_N-based η-carbide phases form over a substantially broader compositional and thermal range than W-based compositions, where η structures are stabilized only when the films are Fe-rich. These trends are rationalized using mixed chemical-potential vs composition phase diagrams that capture the narrow nitrogen chemical-potential stability of η-nitrides. Magnetic measurements reveal that ferromagnetism is induced in Fe-rich Fe_3.54_Mo_2.46_N with a small exchange-bias-like response that is absent in Fe_3_W_3_N-based compositions, highlighting the sensitivity of magnetic behavior to modest deviations from stoichiometry. This work establishes practical thin-film synthesis routes for η-nitride materials and demonstrates how composition can be tuned to access emergent magnetic phenomena in these complex nitrides.

## Graphical abstract

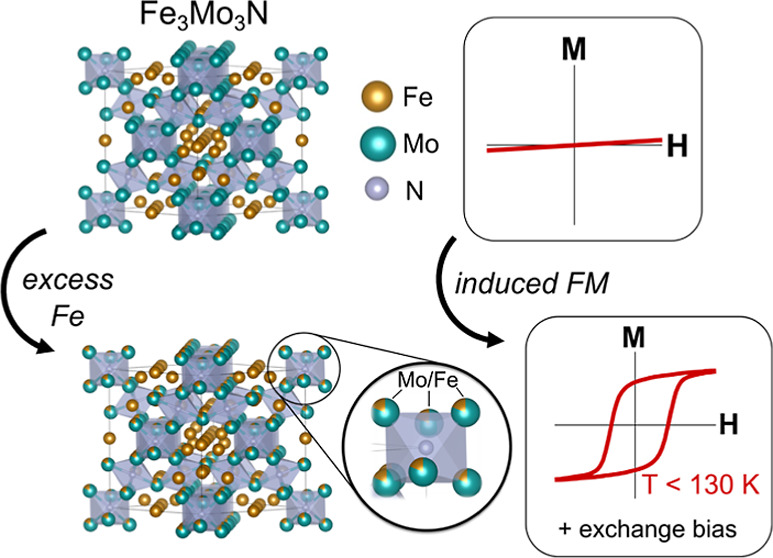

## Introduction

Ternary transition metal nitrides (TMNs) with nitrogen-poor stoichiometries (i.e., involving non-d^0^ TMs) represent an interesting class of materials due to their combination of strong metallic bonding and covalent interactions, giving rise to diverse functional properties ranging from exceptional hardness and thermal stability^1,2^ to superconductivity,^3^ magnetism,^4^ and catalytic activity.^5^ In thin films, we previously showed that nitrogen-rich TMNs can be stabilized under high nitrogen chemical potential (μ_N_), such as plasma-cracked N_2_, where strongly nitriding conditions enable phases that are metastable in the bulk form.^6–9^ We leveraged this strategy to predict and experimentally realize a broad class of new stable and metastable nitrogen-rich transition metal nitride semiconductors.^8,10–12^ In contrast, stabilizing nitrogen-poor TMN thin films presents a fundamentally more challenging problem, as it requires precise control of intermediate μ_N_ in the presence of competing nitride and intermetallic phases. This is amplified in multielement systems, where the number of thermodynamically accessible competing phases grows rapidly. Despite this narrow μ_N_ window, many nitrogen-poor nitrides are thermodynamically stable owing to unique bonding mechanisms, including reductive stabilization, that favor low-nitrogen compositions in multielement systems.^8^ These effects open chemically rich regions of nitride phase space, but realizing them experimentally requires precise control and understanding of synthesis pathways, particularly in the thin-film form.

η-Carbide-type TMNs (referred to as “η-nitrides” in this work) such as Fe_3_W_3_N and Fe_3_Mo_3_N occupy a particularly interesting region of this design space. These compounds are structurally analogous to the well-known η-carbides Fe_3_W_3_C and Fe_3_Mo_3_C, with nitrogen occupying the interstitial sites of the metal framework in place of carbon. This substitution modifies the bonding environment and electronic structure, which directly impacts the mechanical, electronic, and magnetic properties.^13^ While the carbide analogues have been extensively studied as hard coatings,^14,15^ the corresponding nitrides remain relatively underexplored. Importantly, despite extensive bulk investigations, η-nitride thin films have not been reported or systematically studied.

Previous bulk studies on Fe-containing η-nitrides have revealed complex magnetic behavior that is highly sensitive to slight changes in composition.^13,16–22^ Notably, Fe_3_Mo_3_N was found to be located near a quantum critical point, where ferromagnetic (FM) order can be induced from the nonmagnetic ground state through minimal Co-substitution.^18^ These observations position η-nitride materials as a compelling platform for exploring emergent magnetism driven by subtle changes in composition. However, the lack of thin-film synthesis routes and combinatorial approaches has not facilitated the systematic exploration of composition-structure–property relationships in these materials, as metastable or off-stoichiometric regimes are often more easily accessible in thin films.

Here, a combinatorial thin-film approach is employed to synthesize amorphous, compositionally graded Fe–W–N and Fe–Mo–N samples via reactive magnetron cosputtering at room temperature, followed by postdeposition rapid thermal annealing to induce crystallization. Using laboratory X-ray diffraction (XRD) and synchrotron grazing-incidence wide-angle scattering (GIWAXS), the phase evolution of both systems is mapped as a function of composition and annealing temperature. These experimental results are interpreted using calculated chemical-potential vs composition phase diagrams that capture the narrow stability windows of these η-nitrides. Fe_3_Mo_3_N appears to have a wider synthesis window than Fe_3_W_3_N. Moreover, minor stoichiometry deviations yield pronounced changes in magnetic behavior, including inducing ferromagnetism and exchange-bias effects in Fe-rich Fe_3.54_Mo_2.46_N. Together, these results establish practical thin-film synthesis routes for η-nitrides and demonstrate the sensitivity of their magnetic behavior to modest off-stoichiometry.

## Materials and Methods

### Synthesis

Thin film amorphous Fe–W–N and Fe–Mo–N precursors were deposited using radio-frequency (RF) reactive magnetron cosputtering. High-purity elemental 2″ targets of Fe and W or Mo (Kurt J. Lesker, 99.95% purity) were cosputtered in an Ar/N_2_ atmosphere where the Ar and N_2_ flows were set to 8 and 1 sccm, respectively. The RF power of all the source targets was set to 40 W. The base pressure was maintained below 3 × 10^–7^ Torr, and the pressure during deposition was set to 8 mTorr. Films were deposited on 2″ square Si substrates (50.8 × 50.8 mm^2^) coated with a SiN_*x*_ layer of ∼100 nm to prevent any chemical reaction with Si during annealing experiments. The substrates were cleaned in acetone, isopropyl alcohol, and rinsed with DI water. The two magnetrons were positioned in a confocal geometry, and the substrate was maintained stationary to create a composition gradient across the film in one direction (see Figure 1a). The depositions were performed without external heating, considering the self-heating effect from the plasma. After each deposition, the 2″ square combinatorial libraries were first characterized by X-ray fluorescence (XRF) and then cut into stripes along the gradient direction for annealing experiments. Postdeposition annealing was performed in a mini lamp annealer (Advance Riko, MILA-5000) in a flowing nitrogen atmosphere with a flow of 10 slpm. The samples were first heated to 100 °C for 3 min under N_2_ to evaporate surface contaminants and absorbed water and then rapidly ramped up to the desired temperature with a heating rate of 20–30 °C/s. The temperature was maintained for 20 min. At the end, the films were allowed to cool naturally in a N_2_ atmosphere.

**Figure 1:**
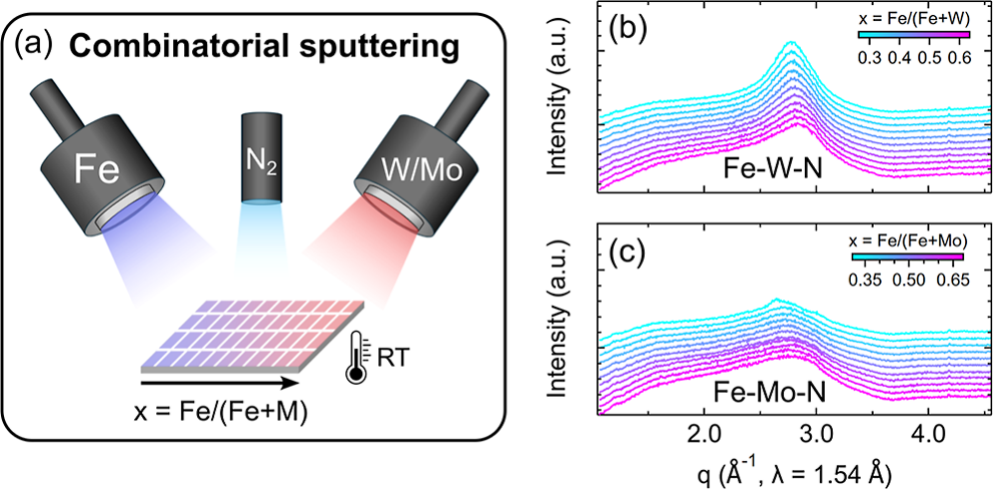
(a) Schematic showing the combinatorial sputtering setup employed in this work. The depositions were performed at room temperature (RT) without external heating. (b) Laboratory XRD patterns of the combinatorial Fe–W–N and Fe–Mo–N as-grown films. A vertical offset is applied for clarity.

### Characterization

The metal-to-metal ratio was characterized by XRF on a Bruker M4 Tornado using an Rh X-ray source operating at 50 kV and 200 μA. XRF spectra were acquired with an exposure time of 120 s and a spot size of 25 μm. An automated stage was used to record spectra across the 2″ square libraries, with a horizontal step of 4 mm and a vertical step of 12.5 mm. The metal atomic ratio in the η-nitrides is defined as *x* = Fe/(Fe + M), where Fe and M represent the atomic fractions of Fe and W or Mo, respectively. Crystalline phases across the films were first screened by laboratory X-ray diffraction (XRD), a Bruker D8 diffractometer equipped with an HI-STAR area detector and operating with a Cu Kα source (λ = 1.54 Å). A mapping stage was used to sequentially acquire diffraction 2D images across the combinatorial samples, with an exposure of 225 s for each point and a 4 mm separation between each point. Samples of interest were further characterized by grazing-incidence wide-angle X-ray scattering (GIWAXS) at the Stanford Synchrotron Radiation Lightsource (SSRL, beamline 11-3), using a 12.7 keV radiation source (λ = 0.97625 Å) and a Rayonix MX225 CCD area detector. The data were collected with a 3° incident angle, a sample-to-detector distance of 150 mm, and a spot size of 50 μm by 150 μm. Structural refinements by the LeBail method were performed with GSAS-II.^23^

Magnetic properties were measured in a Quantum Design DynaCool Physical Property Measurement System (PPMS). ∼5 × 5 mm^2^ samples of interest were cut from the combinatorial stripes and mounted on quartz paddles using GE varnish. DC magnetization was measured upon warming from 2 to 300 K under a field of 5 kOe (0.5 T) applied perpendicular to the sample surface, in both field-cooling (FC) and zero field-cooling (ZFC) modes. Field-dependent magnetization loops were recorded from −10 kOe to 10 kOe at different temperatures. To isolate the contribution of the film from the substrate, a piece of bare substrate was measured in the same configuration and then subtracted from the sample signal, applying a scale factor to account for mass difference. More details about the processing of magnetic data employed in this work can be found in the Supporting Information.

### Calculations

A comprehensive list of Fe–W–N and Fe–Mo–N phases was first assembled from the Materials Project (MP), using the MP API^24^ to filter out entries that were hypothetically generated by crystal structure prediction but have not been experimentally observed, such as the W_2_N_3_ phase (mp-1216242), which along with similar phases overstabilize the ternary hull, thereby removing experimentally confirmed phases like hexagonal WN (mp-991).^6,25^ For each remaining compound, density functional theory calculations were carried out with the Vienna Ab Initio Simulation Package^26^ (VASP) using Pymatgen’s^27^ MPRelaxSet to ensure consistency across calculations. The resulting ground-state energies were subsequently refined through the Materials Project correction scheme^28,29^ to obtain more accurate formation energies for assessing relative phase stability. These energies were then used to construct mixed nitrogen chemical potential–metallic composition phase diagrams by using the Legendre transformed intensive potential given by the following equation

1 $$\phi \left(\mu_{Fe},\mu_{M},\mu_{N}\right)=E\left(N_{Fe},N_{M},N_{N}\right)-\mu_{Fe}N_{Fe}-\mu_{M}N_{M}-\mu_{N}N_{N}$$

where M = Mo, W; μ_*x*_ represents the chemical potential of substance *x*; and *N*_*x*_ is the number of *x* atoms. The lower half-space intersection of this energy hyper-surface in ϕ-μ_M1_-μ_M2_-μ_N_ space is used to obtain the stability domains of each phase. The nitrogen chemical potential stability window is then obtained by projecting this stability domain onto the μ_N_ axis. Finally, the 2-phase and 3-phase coexistence regions are then extracted by identifying domains with shared vertices. Further details of the implementation can be found in the Supporting Information.

## Results and Discussion

### Accessing the η-Nitride Phase through Thermal Annealing of Amorphous Nitride Precursors

Combinatorial deposition of Fe–M–N (M = W, Mo) on Si/SiN_*x*_ substrates without external heating yielded films with the 1:1 Fe/*M* composition near the center of the compositionally graded substrates, as measured with XRF (Figure S1). Figure 1b,c presents XRD patterns collected along the composition gradient of the combinatorial films. The absence of diffraction peaks in both cases indicates that the films are amorphous across the composition range investigated, and no crystalline phases are formed. The broad “humps” observed around *q* = 2.8 Å^–1^ probably correspond to some short-range ordering from metal–nitrogen bonds. As shown in Figure S3a, the as-grown films show metallicity with an electrical resistivity around 100–350 μΩ·cm, which is in the reported range for Fe-based amorphous alloys.^30^ The resistivity only slightly increases with Fe content in amorphous Fe–W–N films, whereas it significantly decreases in Fe–Mo–N.

The effect of postdeposition annealing of the amorphous films was then investigated. Combinatorial strips of Fe–W–N and Fe–Mo–N films were annealed by rapid thermal annealing (RTA) at temperatures ranging from 600 to 900 °C for 20 min under flowing N_2_ (Figure 2a). XRF was performed on postannealed films; no changes in metal compositions were detected, as expected (Figure S2). High-quality synchrotron GIWAXS patterns of films annealed at 800 °C are shown in Figure 2a,b, respectively. The different metal compositions across the graded films result in various phases forming, and the normalized peak intensities for the different phases are plotted versus composition in Figure S4.

**Figure 2:**
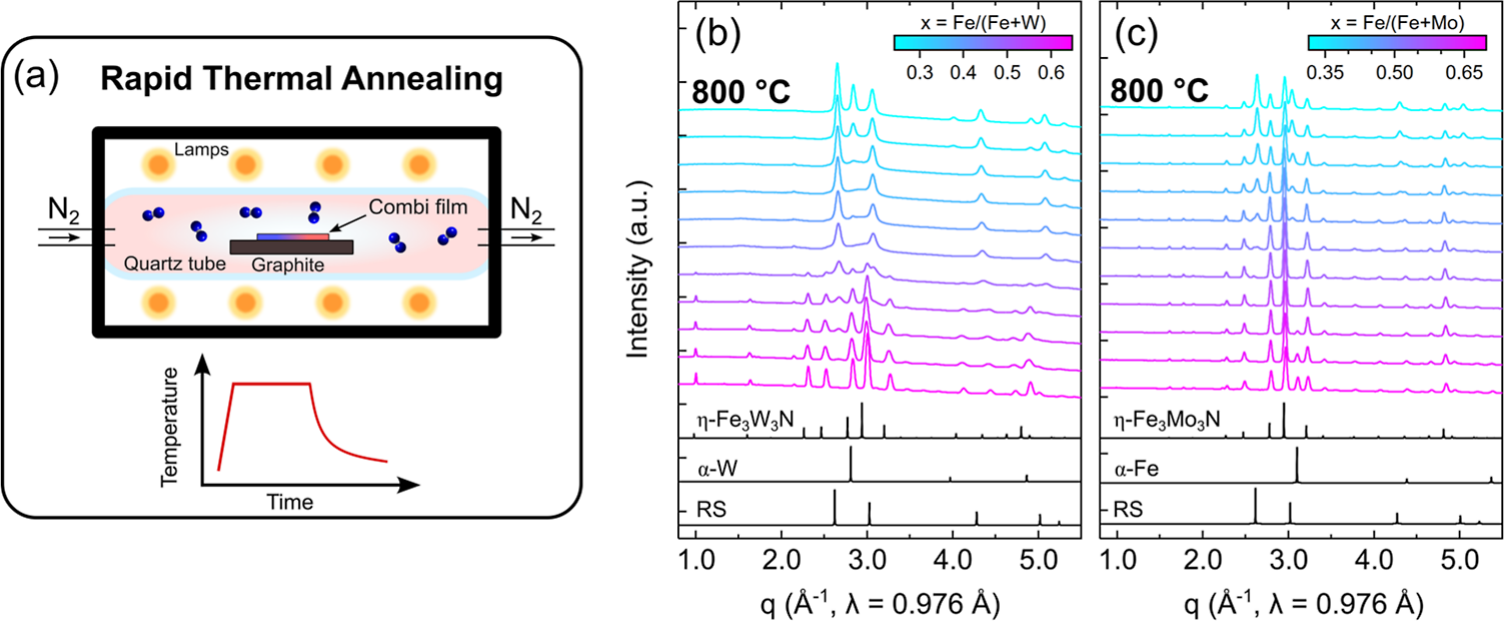
(a) Schematic of the rapid thermal annealing setup employed in this work. The bottom graphic illustrates the typical temperature profile employed for the RTA experiments. Synchrotron GIWAXS patterns of (b) Fe–W–N and (c) Fe–Mo–N films annealed by RTA at 800 °C for 20 min (λ = 0.976 Å). Simulated patterns of relevant phases are added for reference. The term “RS” refers to rocksalt.

First, the Fe–W–N films exhibit a complex evolution of phases as the composition varies (Figure 2b). At W-rich compositions (*x* < 0.5, where *x* = Fe/(Fe + M) and M = Mo, W), the film mostly exhibits a rocksalt-derived face-centered cubic structure (space group *Fm-3d*), which is referred to as “RS” in this work. We note the emergence of the body-centered cubic (bcc) phase of W, labeled α-W for *x* = 0.24–0.28. As the material becomes Fe-rich (*x* > 0.5), it rapidly loses the RS structure and forms an η-nitride phase (nominally Fe_3_W_3_N), as indicated by the characteristic diffraction peaks in GIWAXS patterns. RS + η coexistence is observed from *x* = 0.5–0.61, and then a phase-pure η phase in the narrow window 0.61 ≤ *x* ≤ 0.64, corresponding to a composition closer to Fe_4_W_2_N. This deviation from stoichiometry for the phase-pure composition highlights the flexibility of the η lattice to accommodate extra Fe atoms. In fact, this effect is well known and has been observed in η-carbides based on Fe_3_W_3_C.^31,32^ Another study has even reported the bulk synthesis of nitride Fe_4_W_2_N, in which the authors concluded from Rietveld analysis that the excess Fe substitutes onto W sites.^33^ However, in thin films, detailed site analysis is difficult; our data suggests a compositional tie line over which the η phase accommodates Fe excess without losing its structure. Since stoichiometric Fe_3_W_3_N was successfully synthesized in bulk in previous works,^20,34^ the deviation observed here is unique to our thin-film synthesis approach, in which a metastable RS phase, likely to be WN_*y*_, competes with the formation of the η-nitride at the 1:1 (i.e., *x* = 0.5) stoichiometry.

In the case of Fe–Mo–N films (Figure 2c), the η structure is observed across the whole range of compositions (*x* = 0.31–0.70). An RS phase emerges at Mo-rich compositions and coexists with the η phase. Interestingly, when the film becomes Fe-rich, the bcc phase of Fe (α-Fe) is present. Phase-pure η-nitride is only observed near 1:1 Fe/Mo stoichiometry, in the range *x* = 0.51–0.59. The single-phase region is correlated with a curious increase in electrical resistivity, from 160 to 260 μΩ·cm, before dropping again at higher Fe content (Figure S3). The persistence of Fe_3_Mo_3_N η-based phases at Fe-poor stoichiometry alongside RS or α-Fe peaks suggests partial phase separation, unlike in Fe_3_W_3_N, which only forms at Fe-rich compositions. On the other hand, the composition dependence of the lattice parameter in Fe_3_Mo_3_N shows a slight increase for *x* < 0.5, where RS coexistence is observed, and then a sudden drop around *x* = 0.5 (Figure S5), which was not observed in bulk studies before. This suggests that a reconfiguration of cation sites in the η structure occurs when the Fe content passes the stoichiometric point, provoking an abrupt contraction in the lattice parameter. This notable difference between the two systems indicates that Fe_3_Mo_3_N exhibits a broader synthesis window than Fe_3_W_3_N with a greater accommodation for off-stoichiometry among secondary phases.

### Thermodynamic Insights into η-Nitride Stability

#### Experimental Analysis

To have a complete picture of the synthesis experiments, experimental phase maps of Fe–W–N and Fe–Mo–N are presented in Figure 3a,b, respectively; they summarize the results of XRD/GIWAXS on various combinatorial films annealed from 600 to 900 °C. First, annealing the as-grown amorphous films at 600 °C for 20 min is insufficient to induce crystallization in both systems. For the sample annealed at 700 °C, we observe significant changes for both materials. In Fe–W–N, a rocksalt (RS) phase forms across the whole range of composition *x* = 0.25–0.64, and the η phase starts appearing at Fe-rich compositions along with RS, although the crystallinity is mediocre as suggested by low-intensity XRD peaks, not shown here. The existence of RS in the W-rich region likely originates from WN_*y*_, which is known to form by sputtering, as is often the case for other sputtered binary nitrides.^35–37^ Besides, the composition suggests that a fraction of W is substituted by Fe to form a disordered lattice. Although rocksalt WN is thermodynamically metastable, cation vacancies, nitrogen vacancies, or disorder in general can significantly stabilize it, as is the case for other nitrides and oxides.^38–41^

**Figure 3:**
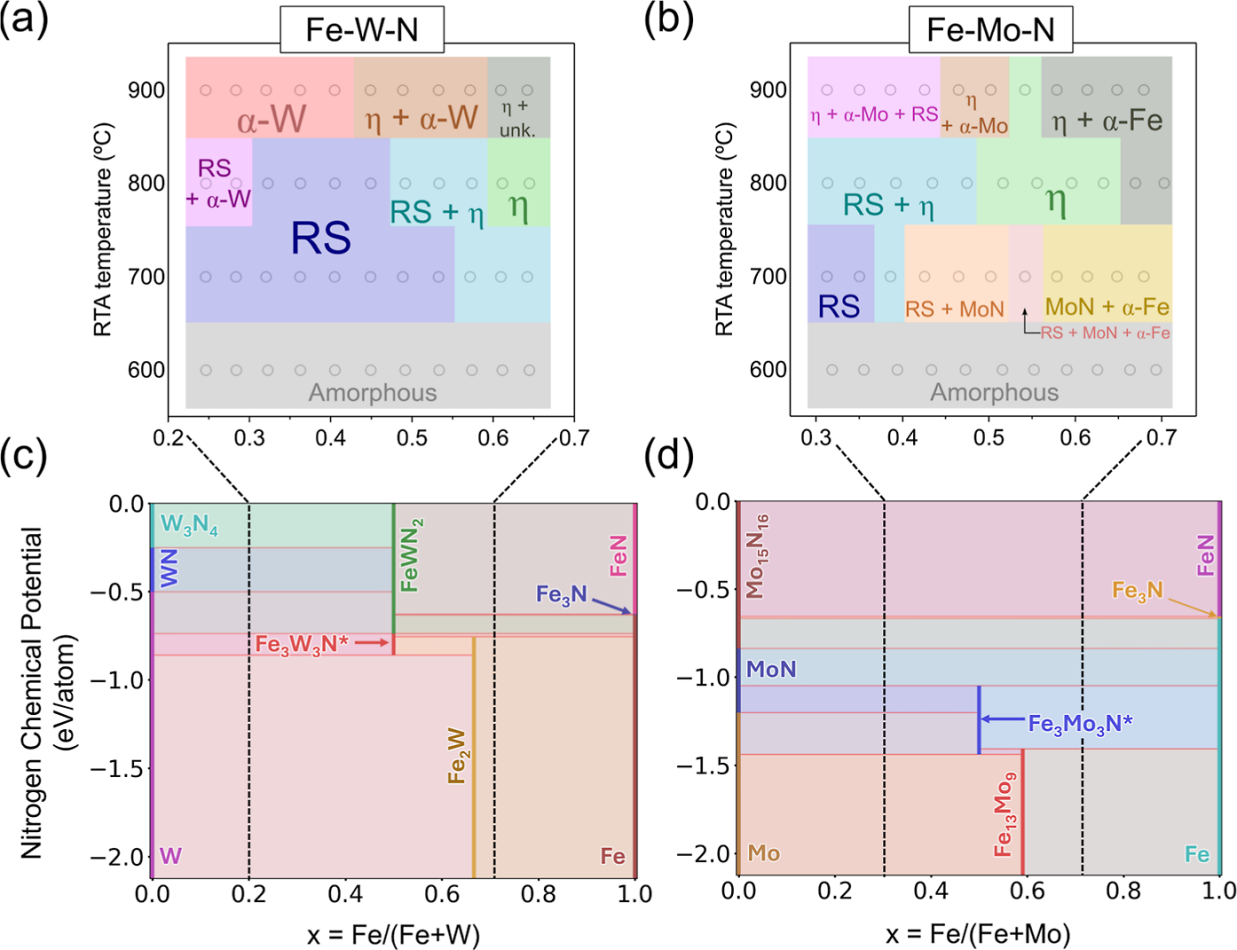
(a,b) Experimental phase maps constructed from composition-dependent XRD results of Fe–W–N and Fe–Mo–N films, respectively, annealed by RTA at temperatures of 600–900 °C. The symbol “η” refers to the η-nitride phase of nominal formula Fe_3_M_3_N (with M = W, Mo) and “RS” stands for rocksalt. (c,d) Nitrogen chemical potential phase diagrams locating the different ground-state phases with respect to metal composition (*x*-axis) and nitrogen chemical potential (*y*-axis), with vertical lines representing single-phase regions, 2D rectangles representing 2-phase regions, and horizontal lines representing 3-phase regions. The dashed lines highlight the regions of composition experimentally investigated in this work.

Annealing Fe–Mo–N films at 700 °C yields a more complex landscape of phase mixtures (Figure 3b). Interestingly, α-Fe forms at Fe-rich compositions, as opposed to the W-based system. A rocksalt MoN phase is observed around *x* = 0.5 and extends to Mo-rich compositions, suggesting the formation of a similar rocksalt-derived structure as in Fe–W–N. Furthermore, hexagonal δ-MoN, which is the thermodynamic ground state in the Mo–N system,^42^ is identified in the range *x* = 0.43–0.68. Overall, a tie line of phase mixture exists across the composition, and the η phase is only observed in a narrow window around *x* = 0.39, along with RS. Those observations suggest that in Fe-rich compositions, Fe and Mo diffuse and segregate to separately form δ-MoN and α-Fe. However, the RS phase is more likely to stabilize at higher Mo content, driven by MoN_*y*_ formation, and therefore, RS predominates in the *x* < 0.5 region. The 800 °C annealing step was discussed above (see Figure 2).

Annealing Fe–W–N films at 900 °C mostly results in the decomposition of the rocksalt phase into bcc W (labeled α-W), as it was already observed at high W content at 800 °C. This is likely due to the higher volatilization of N at 900 °C and is in good agreement with previous studies on the annealing of W–N thin films.^43,44^ Although the η phase exists at Fe-rich compositions, as for 800 °C, it is mixed with α-W as well as an additional unknown phase. Therefore, no phase-pure η is observed at 900 °C. In the Fe–Mo–N system, annealing at 900 °C results in decomposition of the film into bcc-Mo (α-Mo) at Mo-rich compositions. The rapid segregation above 800 °C suggests that excess Fe in the η structure is accommodated through kinetic trapping.

### Computational Analysis

To help understand the differences observed in the two systems, nitrogen chemical potential (μ_N_) phase diagrams for the Fe–W–N and Fe–Mo–N systems were calculated and are presented in Figure 3c,d, respectively. This thermodynamic tool illuminates phase stability regions and areas where multiple phases can coexist at equal μ_N_. We note that the experimental μ_N_ is not trivial to determine as it strongly depends on growth and annealing conditions (including kinetics), so we instead make qualitative comparisons. It also has to be noted that the RS phases observed in experiments are not present in the diagrams as stoichiometric WN, MoN, or FeN in the rocksalt structure are thermodynamically metastable. The Fe–W–N system contains two stable ternary phases: the η-nitride Fe_3_W_3_N and the N-rich hexagonal layered FeWN_2_, which has previously been prepared,^45,46^ but was not observed here. The computed phase diagrams also show various WN_*x*_ and FeN_*x*_ binaries, as well as the intermetallic Fe_2_W. The Fe–Mo–N diagram is relatively similar, except that FeMoN_2_ (isostructural to FeWN_2_) does not appear as it is slightly metastable, even though it has been synthesized by ammonolysis.^47,48^ FeWN_2_ was not experimentally synthesized in this work, but this is likely because ammonia was not used here during postdeposition annealing.

First, the computational phase analysis shows that the stability window of pure Fe_3_W_3_N (described by the height in μ_N_ of the red vertical line at *x* = 0.5) is much narrower in μ_N_ than that of Fe_3_Mo_3_N (0.1 eV/atom versus 0.4 eV/atom, respectively). This suggests that the former may need more precise synthesis conditions to thermodynamically stabilize. In addition, Fe_3_W_3_N is stabilized at a higher μ_N_ (−0.75 eV/atom) than Fe_3_Mo_3_N (−1.05 eV/atom). In general, higher synthesis temperatures correspond to more negative μ_N_, as the chemical potential of gaseous phases scales with μ_N_ = μ_N_^0^ + *RT*ln­(*P*_gas_/*P*°) −*TS*_gas_, where *S*_gas_ is approximately $\frac{3}{2}nk_{B}T$ for monatomic gas or $\frac{5}{2}nk_{B}T$ for diatomic gas. This means that synthesizing Fe_3_Mo_3_N should be more favorable than Fe_3_W_3_N at higher temperatures. This is reflected in the experimental phase maps, as Fe_3_Mo_3_N can be retained in the phase-pure form at 900 °C, whereas Fe_3_W_3_N is not observed in the phase-pure form at higher temperatures. The difference in these stabilizing nitrogen chemical potentials arises from the difference in formation energy between WN (−0.251 eV/atom) and MoN (−0.602 eV/atom).^49^

Fe_3_Mo_3_N is computed to have a 2-phase coexistence with both elemental Fe and Mo over an appreciable μ_N_ window as described by the vertical width of [Fe + Fe_3_Mo_3_N] and [Mo + Fe_3_Mo_3_N] rectangles of 0.36 and 0.24 eV/atom, respectively. In contrast, Fe_3_W_3_N has a small 2-phase coexistence window with W, as described by the width of the [W + Fe_3_W_3_N] rectangle in red, as well as an extremely narrow 2-phase coexistence with Fe, as described by the very thin rectangle [Fe + Fe_3_Mo_3_N] above Fe_2_W. This is in strong agreement with the experimental maps in which a large coexistence of Fe_3_Mo_3_N with both α-Fe or α-Mo was observed, as opposed to Fe_3_W_3_N, in which coexistence with α-W exists around *x* = 0.5, but no coexistence with α-Fe was observed, in the detection limits of laboratory XRD. These findings suggest that the synthesis of Fe_3_W_3_N requires greater compositional and chemical potential control.

The decomposition into α-W or α-Mo at high temperature can be understood as follows: at fixed pressure, the experimental μ_N_ is expected to decrease with temperature. However, since both Fe_3_W_3_N and Fe_3_Mo_3_N persist at 900 °C, this could indicate that μ_N_ is still large enough to stabilize the η phases. We note that in Fe–W–N, no phases located above Fe_3_W_3_N in μ_N_ are observed in the experiments, as opposed to Fe–Mo–N, in which hexagonal MoN is obtained at 700 °C and then vanishes at 800 °C to favor Fe_3_Mo_3_N, which implies that μ_N_ falls below −1.30 eV, where the MoN window ends.

### Crystallographic Analysis of η-Nitride Films

To further characterize the crystal structure of the η-nitride phase in films annealed at 800 °C, refinements of GIWAXS patterns using the LeBail method were performed at compositions where the η phase is present without distinct secondary phases and shows maximum peak intensities in XRD (Figure S4). Thus, Fe_3.84_W_2.16_N (corresponding to *x* = 0.64) and Fe_3.54_Mo_2.46_N (i.e., *x* = 0.59) compositions were selected. Results of the fits are presented in Figure 4a,b. The corresponding detector images converted to reciprocal space are displayed in Figure 4c,d. The good continuity of diffraction rings confirms that both films are polycrystalline without any preferential orientations. The GIWAXS patterns were fitted to the η-nitride structure of Fe_3_W_3_N and Fe_3_Mo_3_N in the space group *Fd*3̅*m* (Figure 4e).

**Figure 4:**
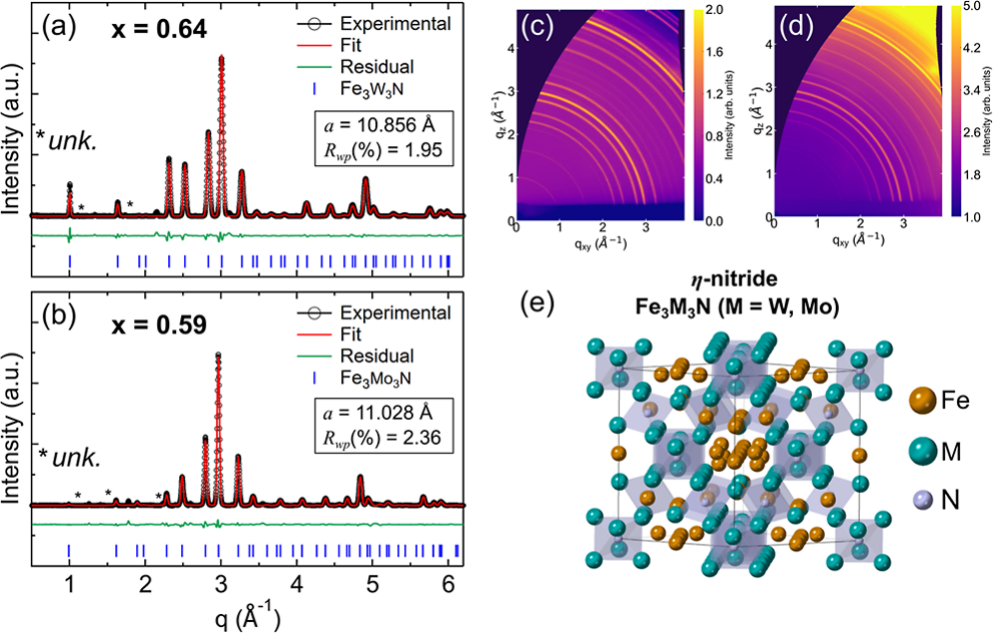
(a,b) LeBail refinements of synchrotron GIWAXS data and (c,d) the corresponding GIWAXS detector images converted into reciprocal space of (a,c) Fe_3.84_W_2.16_N (*x* = 0.64) and (b,d) Fe_3.54_Mo_2.46_N (*x* = 0.59). The label “*unk*.” refers to unknown phases. (e) Crystal structure of η-nitride Fe_3_ *M*_3_N (M = W, Mo).

For Fe_3.84_W_2.16_N (Figure 4a), the LeBail refinement yields a lattice parameter *a* = 10.856 Å with a profile weighted *R*-factor *R*_wp_ = 1.95%. It is interesting to note that the lattice parameter is significantly smaller than the value reported in the literature for Fe_3_W_3_N powder prepared by ammonolysis (*a* = 11.11 Å).^34^ This could be due to relaxation occurring during rapid annealing or simply due to the Fe-rich stoichiometry, which is closer to Fe_4_W_2_N in this case. In fact, the lattice parameter of Fe_4_W_2_N powder has already been reported and is slightly smaller than Fe_3_W_3_N (*a* = 11.08 Å).^50^ However, this reduction by only 0.3% from Fe_3_W_3_N to Fe_4_W_2_N in bulk materials cannot explain the significant difference observed in our thin films. Another possibility could be due to the nitrogen stoichiometry. Many materials are also stable in the η structure with N-deficient 6:6:1 stoichiometry and a smaller lattice parameter, such as Fe_6_W_6_C or Fe_6_W_6_N (Figure S8).^32,51,52^ The absence of peak splitting in our GIWAXS patterns rules out the coexistence of both Fe_3_W_3_N and Fe_6_W_6_N phases. Despite the good phase purity, we note the presence of a low-intensity peak at *q* = 3.1 Å^–1^ (magnified in Figure S6), corresponding to (110) reflection of α-Fe, which was not detected by laboratory XRD. Two additional low-intensity peaks are observed at low *q*, marked with asterisks (*), suggesting that other small crystalline impurities are present. The low amplitude of these peaks, relative to the main peaks, could suggest that these are limited to the surface and could come from oxides formed during annealing, especially due to the excess of Fe. However, those peaks could not be attributed to any oxide phase with certainty.

The refinement of Fe_3.54_Mo_2.46_N (*x* = 0.59) shown in Figure 4b yields a lattice parameter *a* = 11.028 Å with *R*_wp_ = 2.36%. The value of *a* is close to the reported value for bulk Fe_3_Mo_3_N, although slightly smaller (*a* = 11.067 Å).^22^ This difference is likely due to the excess Fe, which reduces the lattice size, as discussed previously. Although the fit is of good quality and shows decent phase purity, a few low-intensity peaks from unknown phases are detected at low *q* (*) and are likely to come from surface oxides, as in the W system, but do not match with α-Fe. In conclusion, these refinements confirm that both Fe_3.84_W_2.16_N and Fe_3.54_Mo_2.46_N films show good phase purity of η-nitride, with respect to a small amount of unknown impurities, which we associate with surface oxides. Nevertheless, these films can still be considered “phase-pure” in bulk.

### Dependence of Ferromagnetism upon Subtle Composition Changes

The η-carbide-type compounds containing magnetic elements are known to exhibit interesting and complex itinerant-type magnetism in the bulk form arising from the geometrical frustration inherent to the η-carbide-type structure, and the delicate balance between metal–metal and metal–nitrogen bonding, which makes these systems highly responsive to changes in composition.^53,54^ Thin films offer an additional degree of control over composition, disorder, and defect concentration, which may further tune magnetic interactions. Motivated by these considerations, we investigate in the following sections how deviations from stoichiometry and phase competition influence the magnetic response of Fe_3_W_3_N and Fe_3_Mo_3_N films annealed at 800 °C. Fe-rich compositions exhibiting phase-pure η as observed by GIWAXS are compared with near-stoichiometric films (*x* ∼ 0.5) where secondary RS phases are present (as highlighted in Figure S7). The magnetic data presented in the following sections are normalized per formula unit of Fe_3_W_3_N or Fe_3_Mo_3_N to aid in analysis at low temperatures.

### Magnetic Susceptibility

Figure 5 shows the temperature-dependent magnetic DC susceptibility χ = *M*/*H* (where *M* is the magnetization and *H* is the applied field) measured under zero-field cooled (ZFC) and field-cooled (FC) conditions with a constant field of 5 kOe for η-nitride films at Fe-rich composition as well as near stoichiometry. Both systems display Curie–Weiss-like paramagnetic behavior at high temperatures with a monotonic decrease of χ upon warming. In the Fe–W–N system (Figure 5a), the susceptibility shows a smooth transition around 130 K at both compositions, indicating the onset of ferromagnetic (FM) ordering, which aligns with the observations reported in bulk Fe_3_W_3_N.^20^ The Fe-rich sample Fe_3.84_W_2.16_N (*x* = 0.64) shows a significant vertical offset at high temperatures, which can be ascribed to FM impurities with a high Curie temperature, most likely coming from the α-Fe secondary phase (see Figure S6). In the near-stoichiometric sample Fe_3.18_W_2.82_N (*x* = 0.53), the slightly negative χ at high temperatures could be due to a weak diamagnetic background, even after substrate subtraction.

**Figure 5:**
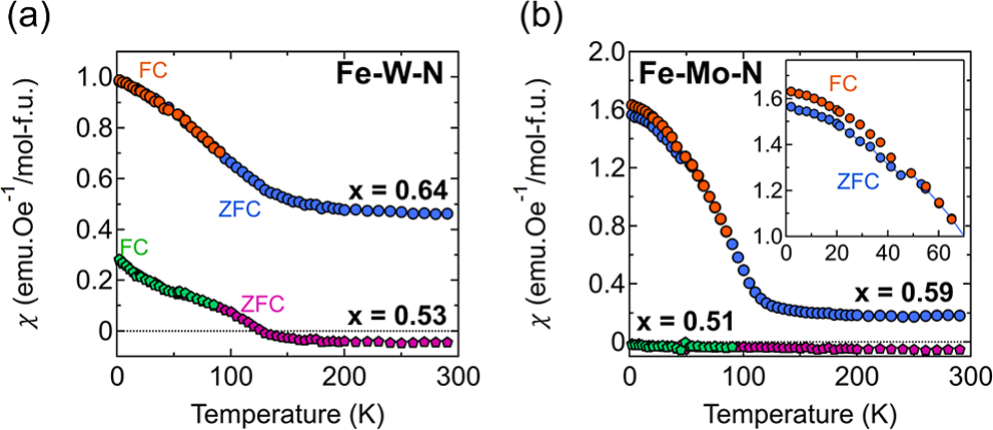
Temperature-dependent magnetic susceptibility of η-nitride films (a) Fe_3.84_W_2.16_N (*x* = 0.64) and Fe_3.18_W_2.82_N (*x* = 0.53) and (b) Fe_3.54_Mo_2.46_N (*x* = 0.59) and Fe_3.06_Mo_2.94_N (*x* = 0.51). The susceptibility was measured while heating up from 2 K, under an applied field of 5 kOe (0.5 T) in ZFC and FC conditions. The FC traces are only displayed at low temperatures for clarity. In the case of Fe_3.54_Mo_2.46_N, the inset magnifies the small ZFC/FC splitting at low temperature. Although the compositions differ in stoichiometry, the magnetic susceptibility is normalized per mole of formula unit of Fe_3_W_3_N and Fe_3_Mo_3_N, respectively.

The Fe–Mo–N system displays qualitatively different behavior (Figure 5b). In Fe_3.54_Mo_2.46_N (*x* = 0.59), a pronounced transition in χ is observed around the same transition temperature as in Fe–W–N. The susceptibility sharply increases and seems to saturate around 1.6 emu Oe^–1^/mol-f.u. at 2 K, which is much higher than in the W system and indicates a more pronounced FM ordering. This FM transition is accompanied by a small bifurcation between ZFC and FC susceptibility curves below approximately 50 K (Figure 5b inset). While subtle, this splitting could suggest the presence of competing interactions at low temperature, potentially due to site disorder, domain pinning, or coupling between multiple magnetic phases. Although no crystalline α-Fe or other ferromagnetic impurities are detected in GIWAXS patterns at this composition, the nonzero χ at high temperatures (∼0.2 emu Oe^–1^/mol-f.u.) suggests minor paramagnetic impurities, potentially from surface oxides. As opposed to Fe–W–N, the near-stoichiometric film Fe_3.06_Mo_2.94_N (*x* = 0.51) shows no magnetic ordering across the entire temperature range studied, consistent with prior neutron diffraction studies on bulk samples,^18^ confirming that FM behavior in this system is induced by tuning the stoichiometry, rather than intrinsic to the Fe_3_Mo_3_N η-nitride structure.

### Field-Dependent Magnetization

Figure 6 shows the field-dependent magnetization loops at different temperatures. The Fe_3.06_Mo_2.94_N film is not shown as it shows no magnetic ordering. In Fe–W–N, the presence of hysteresis in both samples confirms the FM ordering. In Fe_3.18_W_2.82_N (Figure 6a), the hysteresis vanishes beyond 100 K, and the magnetization becomes slightly negative, likely an artifact stemming from low signal and substrate subtraction. This corroborates the absence of an α-Fe impurity at this near-stoichiometric composition, illustrated by the saturation magnetization (*M*_s_) and the coercive field (*H*_*c*_) reaching zero at high temperatures (Figure 7a–c). *H*_c_ and *M*_s_ linearly increase below a Curie temperature (*T*_c_) estimated at 130 K and, respectively, reach 223 Oe and 0.27 μ_B_/f.u. at 2 K. In Fe_3.84_W_2.16_N, the hysteresis persists at 300 K, confirming that α-Fe ferromagnetic impurity dominates at high temperatures. We note that the normalization per formula unit of Fe_3_W_3_N is not valid in this temperature range. The emergence, temperature dependence, and disappearance of hysteresis near the ordering transition indicate that the dominant low-temperature magnetic behavior is associated with the η-nitride phase. The shape of the hysteresis loop (or “squareness”) is more pronounced below 150 K, suggesting that the FM ordering of the η phase dominates over the impurity. The coercive field (Figure 7a) is 137 Oe at 2 K and decreases with temperature as expected, before a sudden jump to 232 Oe around the transition, where FM from α-Fe takes over, which is in the typical range of coercivity observed in α-Fe thin films after annealing.^55^ The magnitude of *H*_c_ in the η phase is relatively small and suggests the material is a soft ferromagnet.

**Figure 6:**
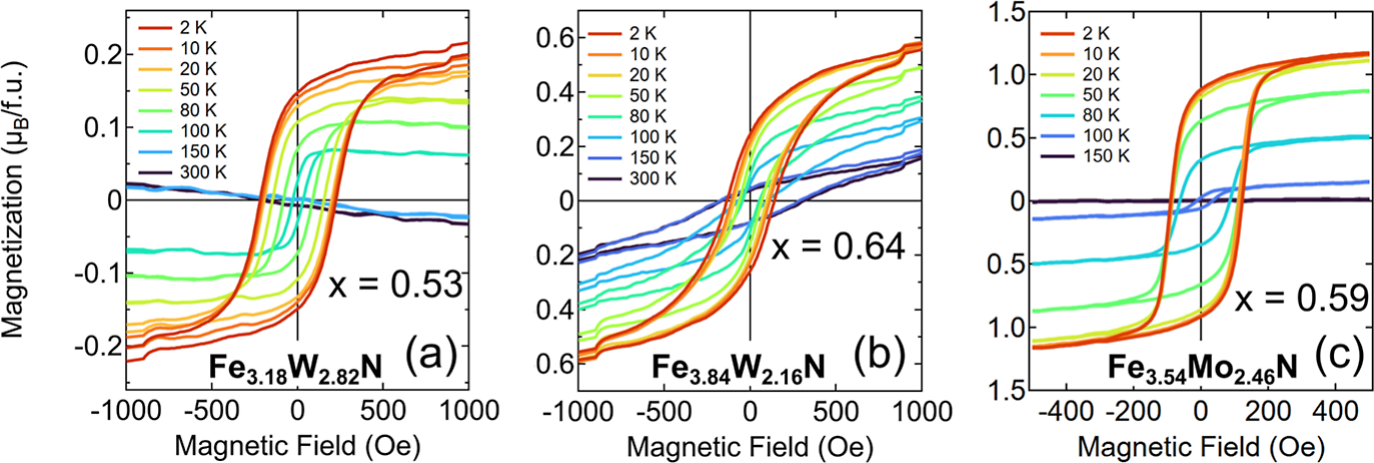
Magnetization loops of selected η-nitride films at different temperatures: (a) Fe_3.18_W_2.82_N (*x* = 0.53), (b) Fe_3.84_W_2.16_N (*x* = 0.64), and (c) Fe_3.54_Mo_2.46_N (*x* = 0.59).

**Figure 7:**
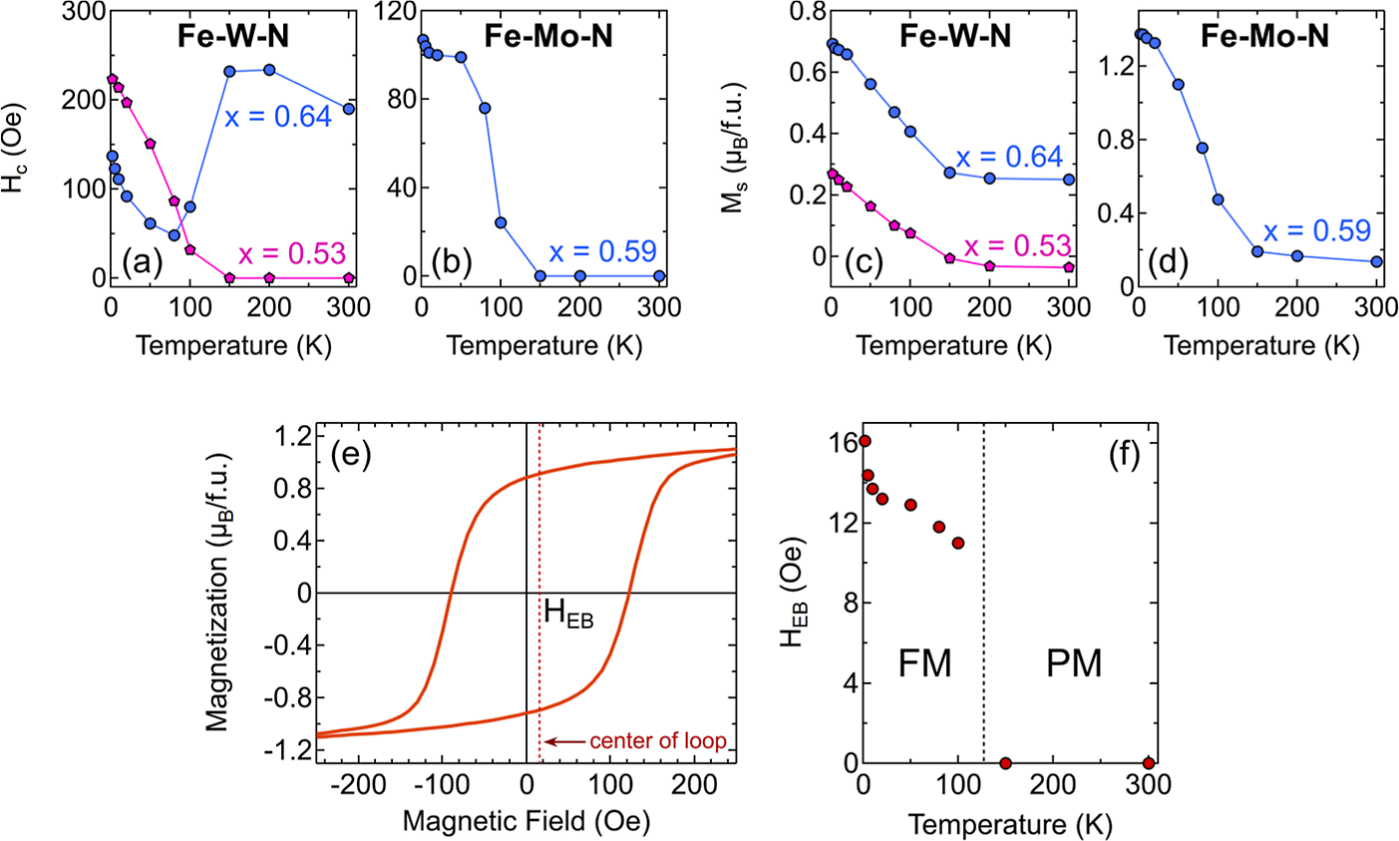
Temperature-dependent coercive field (*H*_c_) and saturation magnetization (*M*_s_) of (a,c) Fe_3.84_W_2.16_N (*x* = 0.64) and Fe_3.18_W_2.82_N (*x* = 0.53) and (b,d) Fe_3.54_Mo_2.46_N (*x* = 0.59). *M*_s_ values are extracted at 10 kOe (see Figure S10). (e) Magnetization loop of Fe_3.54_Mo_2.46_N recorded at 2 K, highlighting the horizontal shift of the loop. *H*_EB_ refers to the exchange-bias (EB) field. (f) Temperature dependence of *H*_EB_ in Fe_3.54_Mo_2.46_N. FM and PM refer to ferromagnetic and paramagnetic regions, respectively.

In Figure 6c, Fe_3.54_Mo_2.46_N shows clear hysteresis with greater squareness than Fe–W–N films. The hysteresis region shrinks with temperature until it completely vanishes beyond 100 K, confirming the FM transition observed in susceptibility measurements. The coercive field at 2 K is measured at 107 Oe and decreases with temperature within the FM regime, as shown in Figure 7b. Moreover, it shows a two-step trend by first reaching a plateau around 50 K and then rapidly dropping to zero while crossing the FM transition upon cooling. This unusual behavior could indicate the presence of two magnetic populations. The *M*_s_ extracted at a higher field reaches 1.37 μ_B_/f.u. (0.46 μ_B_/Fe), assuming the formula unit of Fe_3_Mo_3_N, and its temperature dependence, shown in Figure 7, aligns well with the transition observed in the magnetic susceptibility. Since the stoichiometric film does not show FM, it is interesting to estimate the magnetization per excess of Fe in Fe_3.54_Mo_2.46_N. If the offset at 300 K (0.14 μ_B_/f.u.) due to impurity is subtracted, *M*_s_ at 2K becomes 1.23 μ_B_/f.u. Since there are 0.54 Fe atoms in excess per formula unit, the resulting *M*_s_ is 2.28 μ_B_ per excess Fe atom, which is below the solubility limit determined in the Fe_3_W_3_N system. This value is close to the magnetic moment of α-Fe (2.2 μ_B_), suggesting some itinerant magnetism.

Overall, the η-nitride films investigated here exhibit soft ferromagnetic behavior with a Curie temperature around 130 K, characterized by modest magnetization and low coercivity (on the order of ∼100 Oe at low temperature). The magnetic properties of the four different compositions investigated along with their structural and chemical properties are summarized in Table 1. These values are significantly smaller than those of high-performance nitride-based candidates for permanent magnets such as Fe_16_N_2_ or Sm–Fe–N compounds, which typically display substantially larger magnetization and coercivity.^56,57^ Furthermore, the soft ferromagnetism observed in the Fe_3_M_3_N films in this work can be compared to hexagonal Fe_3_N, which has an equal Fe/N ratio and exhibits relatively small coercivity in thin films. However, it is interesting to note that the saturation magnetization in Fe_3_N films is typically higher, with a *T*_c_ above room temperature.^58^ The more complex nature of the η structure and the presence of a nonmagnetic W or Mo sublattice could disrupt the exchange interactions between magnetic Fe ions, leading to a reduction in the Curie temperature and a decrease in the overall magnetization. While Fe_3_Mo_3_N shows no magnetic ordering near stoichiometry, a modest excess of Fe induces itinerant-type ferromagnetism while maintaining the η structure. It is important to note that this composition-induced ferromagnetism in Fe_3.54_Mo_2.46_N exists with a Curie temperature similar to Fe_3_W_3_N (∼130 K), confirming it is not due to Fe nanoclusters or inclusions, which should induce room-temperature ferromagnetism. The same argument applies to Fe–N phases, which typically exhibit *T*_C_ above room temperature. The recovery of ferromagnetism could suggest that the modest excess of Fe substitutes for a fraction of Mo, reducing the negative exchange interactions and therefore inducing a net moment. In other words, the magnetic softness and great sensitivity to composition highlight a different opportunity: η-nitrides serve as a platform for investigating composition-driven magnetic instabilities and frustration in nitrogen-poor transition-metal systems. In this context, their value lies less in maximizing magnetic performance and more in enabling controlled exploration of correlated and emergent magnetic behavior in structurally robust nitride frameworks.

**I tbl1:** Summary of Structural and Magnetic Properties of the Key Compositions Investigated

thin film composition	dominant phase	lattice parameter (Å)	minor secondary phase by XRD	magnetic response	*T*_C_ (K)	*H*_c_@2K (Oe)	*M*_s_@2K (μ_B_/f.u.)
Fe_3.18_W_2.82_N_*y*_	η	10.873	RS	FM	∼130	223	0.27
Fe_3.84_W_2.12_N_*y*_	η	10.856	α-Fe	FM (with Fe contribution)	∼130	137	0.69
Fe_3.06_Mo_2.94_N_*y*_	η	11.051	RS	nonmagnetic	N/A	N/A	N/A
Fe_3.54_Mo_2.46_N_*y*_	η	11.028	none	FM	∼130	107	1.37

### Exchange-Bias-Type Behavior in Fe_3.54_Mo_2.46_N

A small shift in the hysteresis loop is observed in Fe_3.54_Mo_2.46_N at low temperatures, as highlighted in Figure 7e, suggesting exchange-bias (EB)-type behavior with an EB field (*H*_EB_) of approximately 16 Oe at 2 K. While small in magnitude, this effect systematically changes with temperature and is reproducible across different measurements. Such an effect was not observed in comparable Fe_3.84_W_2.12_N films. EB typically arises from interfacial coupling between different magnetic regions, such as FM, AFM, and magnetically disordered phases.^59–61^ In the present case, the origin of this EB behavior cannot be unambiguously identified. It may reflect magnetic heterogeneity associated with compositional disorder, nanoscale phase separation, or competing magnetic interactions within the Fe-rich η-nitride stability window.

Although bulk Fe_3_Mo_3_N was first categorized as antiferromagnetic, following studies confirmed the absence of magnetic long-range order down to 10 K.^18,62^ The same authors showed that Fe_3_Mo_3_N is located near a quantum critical point and that FM order can easily be induced by small chemical substitutions of ferrous elements such as Co.^18,22^ Our observation that Fe_3.06_Mo_2.94_N films remain nonmagnetic while modest excess Fe induces FM is consistent with this picture. In this context, the EB-like response observed only in Fe-rich films may reflect magnetic heterogeneity arising from proximity to this instability, which is enabled by off-stoichiometric compositions accessible to our thin-film synthesis approach. In thin films, modest Fe excess may be accommodated either through substitutional disorder or nanoscale phase heterogeneity within the η-nitride stability window. While the microscopic origin of the induced ferromagnetism and exchange-bias-like response cannot be resolved from the present data, these results highlight the pronounced sensitivity of Fe_3_Mo_3_N magnetism to small deviations from stoichiometry.

## Conclusion

In summary, using a combinatorial synthesis approach and postdeposition RTA in the 600–900 °C range, we demonstrate that η-nitride Fe_3_W_3_N and Fe_3_Mo_3_N can be stabilized as thin films, and we identify synthetic windows to achieve phase-pure products. While η-Fe_3_Mo_3_N forms across a wide composition range, η-Fe_3_W_3_N is only obtained at Fe-rich compositions. Thermodynamic calculations showing significant differences in stability windows between the Fe–Mo–N and Fe–W–N systems rationalize the more precise synthesis conditions needed to synthesize thin films of Fe_3_W_3_N. A sharp lattice parameter contraction beyond stoichiometry in Fe_3_Mo_3_N indicates a structural response to excess Fe, pointing to either a rearranged Fe-rich η phase or nanoscale phase separation within the η-nitride stability field. Magnetic measurements on films reveal ferromagnetic ordering in Fe_3_W_3_N around *T*_C_ ∼130 K. Interestingly, a modest excess of Fe induces clear ferromagnetism in Fe_3_Mo_3_N around the same temperature, as well as a small exchange-bias effect, suggesting competition between different magnetic phases or sublattices. These findings emphasize the sensitivity of η-nitrides to off-stoichiometry, reveal their phase stability and structural flexibility in thin-film form, and demonstrate how careful synthetic control enables access to emergent magnetic behavior in nitrogen-poor nitrides.

## Supplementary Material



## Notes

Additional data for XRF, electrical resistivity, X-ray diffraction, magnetometry. Additional method details on the calculations of mixed chemical potential-composition phase diagrams and data processing for magnetic measurements (PDF)
