# Supplementary material for: Thin-Film Stabilization and Magnetism of η‑Carbide-Type Iron Nitrides
Source: Chem Mater. 2026 Jun 17;38(13):6723–32. doi: 10.1021/acs.chemmater.6c00901 (PMC13374006; doi:10.1021/acs.chemmater.6c00901)
Supplement: Supplementary file 1 [file cm6c00901_si_001.pdf]

Thin-Film Stabilization and Magnetism of η-Carbide Type Iron Nitrides

Baptiste Julien[1]*, Abrar Rauf[2], Liam A. V. Nagle-Cocco[3], Rebecca W. Smaha[1], Wenhao Sun[2], Andriy Zakutayev[1], Sage R. Bauers[1]

1. *Materials Science Center, National Laboratory of the Rockies, Golden, CO 80401, United States.*
2. *Department of Materials Science and Engineering, University of Michigan, Ann Arbor, MI 48109-1079, United States.*
3. *Stanford Synchrotron Radiation Lightsource, Stanford University, Menlo Park, CA 94025, United States*

* *email:* baptiste.julien@nlr.gov

Supplementary Materials

I. X-ray Fluorescence (XRF)

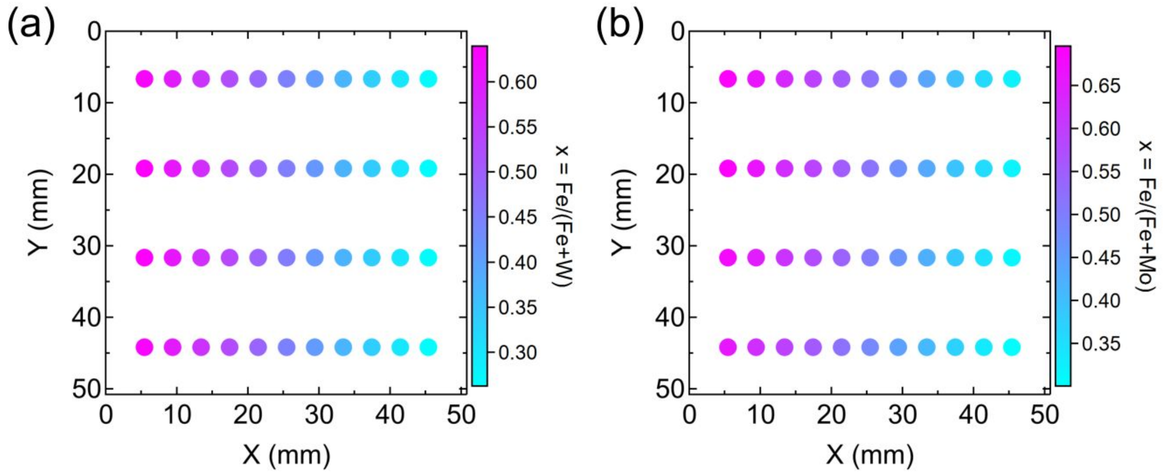

Figure S1. XRF maps of as-grown combinatorial 2” libraries of (a) Fe-W-N and (b) Fe-Mo-N. The metal atomic ratio x = Fe/(Fe+*M*) (*M* = W, Mo) is extracted at each position on the 2” library which is divided into 4 rows and 11 columns (44 points total). In the confocal setup used in this work, a horizontal composition gradient is obtained whereas the vertical gradient is minimal. Thus, each row can be cleaved and used separately for specific experiments.

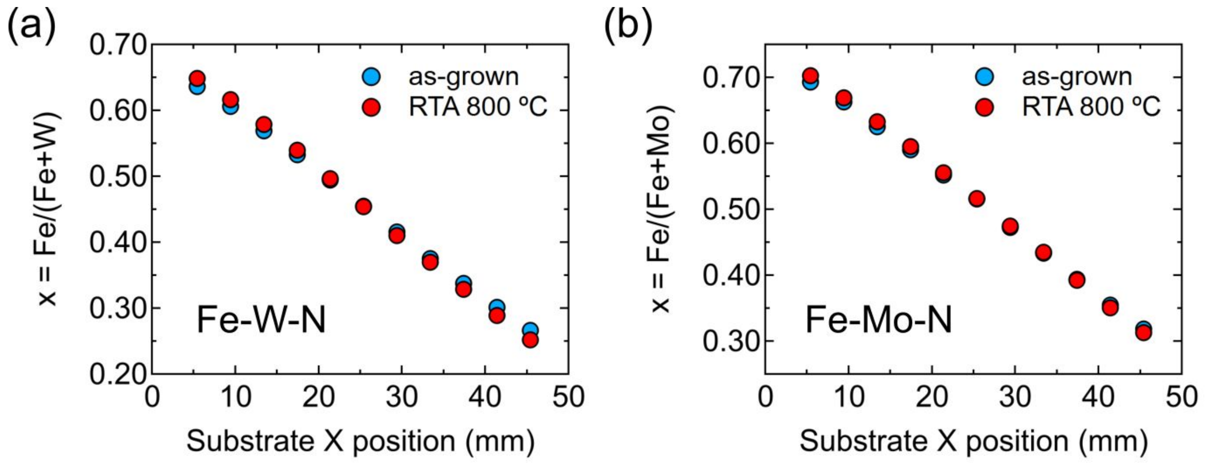

Figure S2. Comparison of metal composition between as-grown and annealed films in (a) Fe-W-N and (b) Fe-Mo-N. The difference is minimal, showing that no horizontal diffusion occurs during annealing.

II. Electrical Resistivity

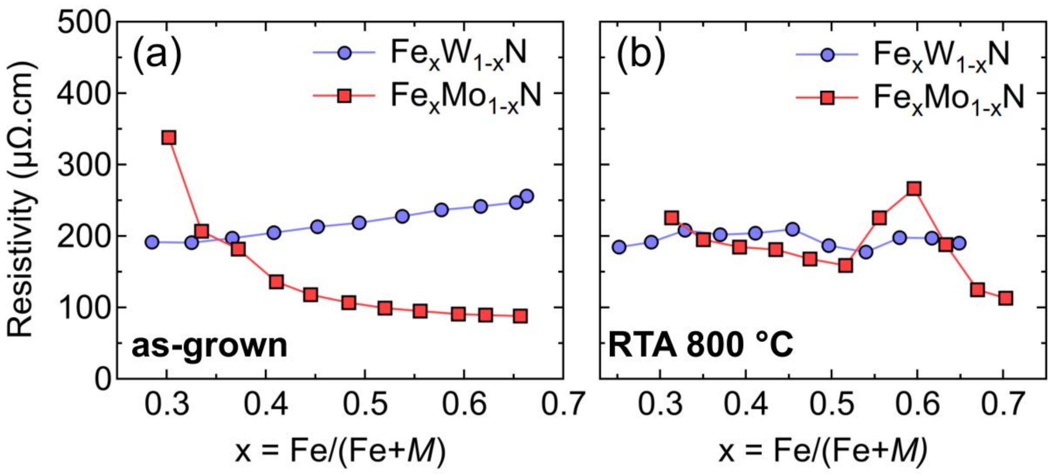

Figure S3. Composition-dependent resistivity of (a) as-grown $Fe_xW_{1-x}N$ and $Fe_xMo_{1-x}N$ amorphous films and (b) films annealed at 800 °C. The resistivity was calculated from the sheet resistance measured by four-point probe method and from the local thickness extracted from XRF.

III. Structural Characterization

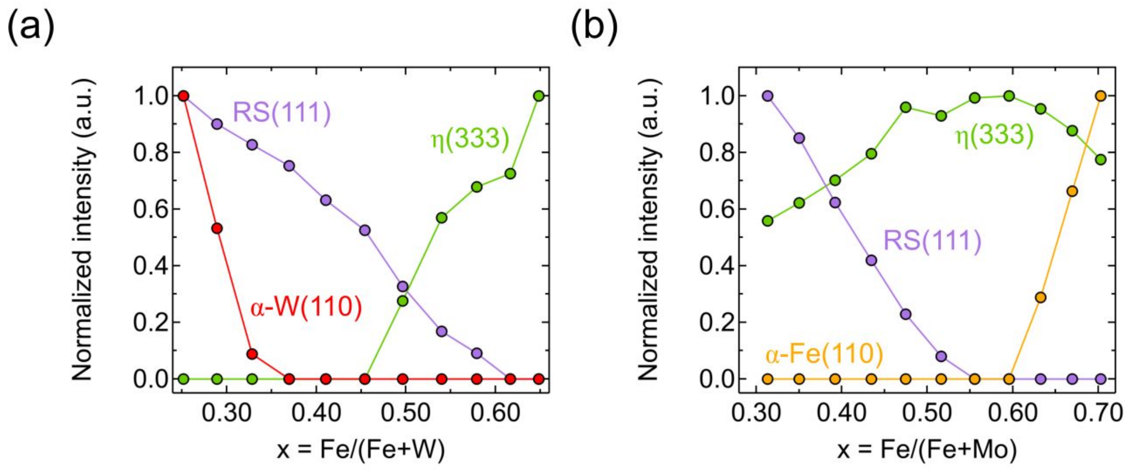

Figure S4. Evolution with composition of XRD peak normalized intensities of the different phases identified in (a) $Fe_xW_{1-x}N$ and (b) $Fe_xMo_{1-x}N$ films annealed at 800 °C. The labels 'η' and 'RS' refer to the η-nitride phase and the rocksalt phase, respectively.

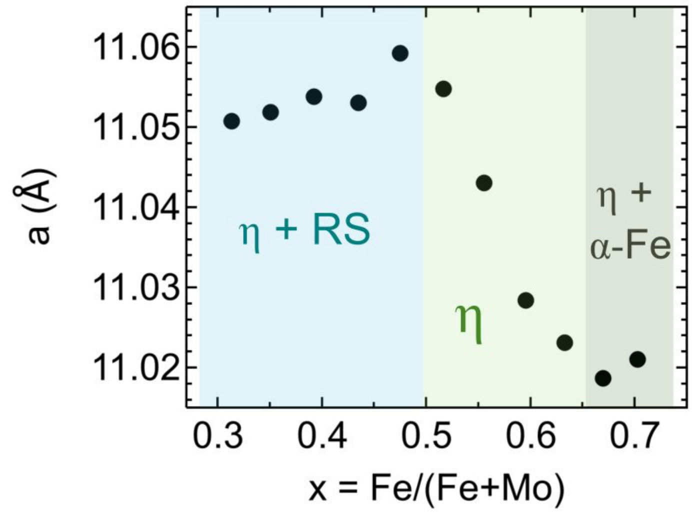

Figure S5. Composition dependance of the η lattice parameter in $Fe_xMo_{1-x}N$ film annealed at 800 °C. A sudden drop in the lattice parameter occurs in the phase-pure region.

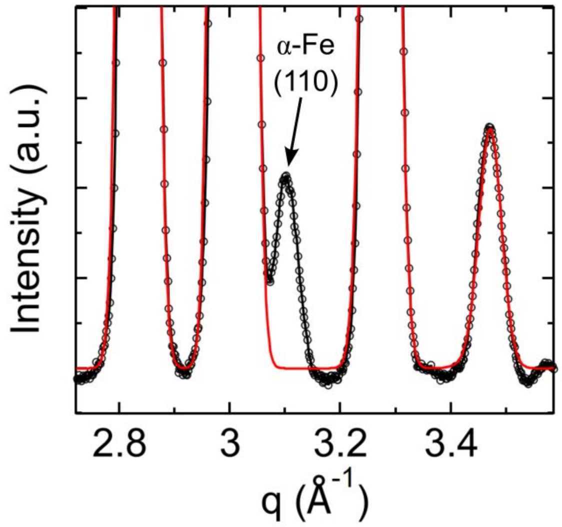

Figure S6. GIWAXS patterns of η-Fe_3W_3N and LeBail fit magnifying around the low-intensity reflection at q = 3.1 Å^{-1} corresponding (110) of α-Fe secondary phase.

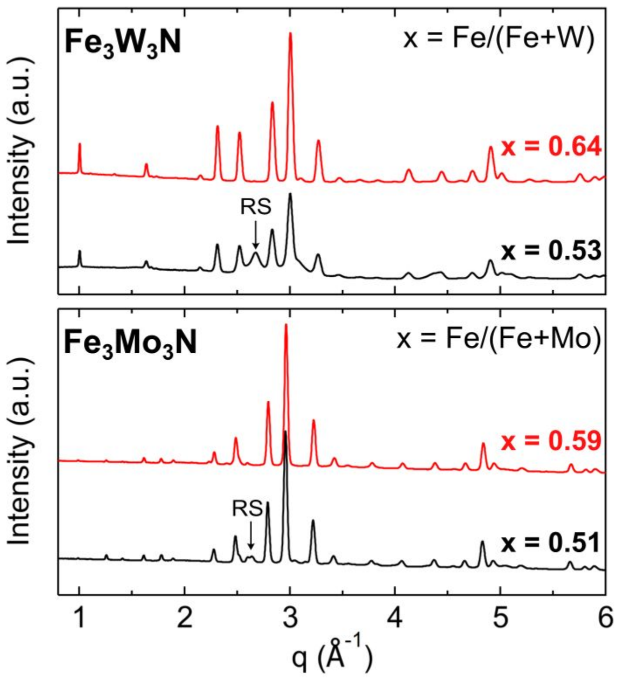

Figure S7. GIWAXS patterns of $Fe_xW_{1-x}N$ and $Fe_xMo_{1-x}N$ films annealed at 800 °C comparing two different metal-to-metal ratios: single-phase η at Fe-rich compositions (x > 0.5) and near-stoichiometry (x ~ 0.5) which exhibit rocksalt (RS) impurities.

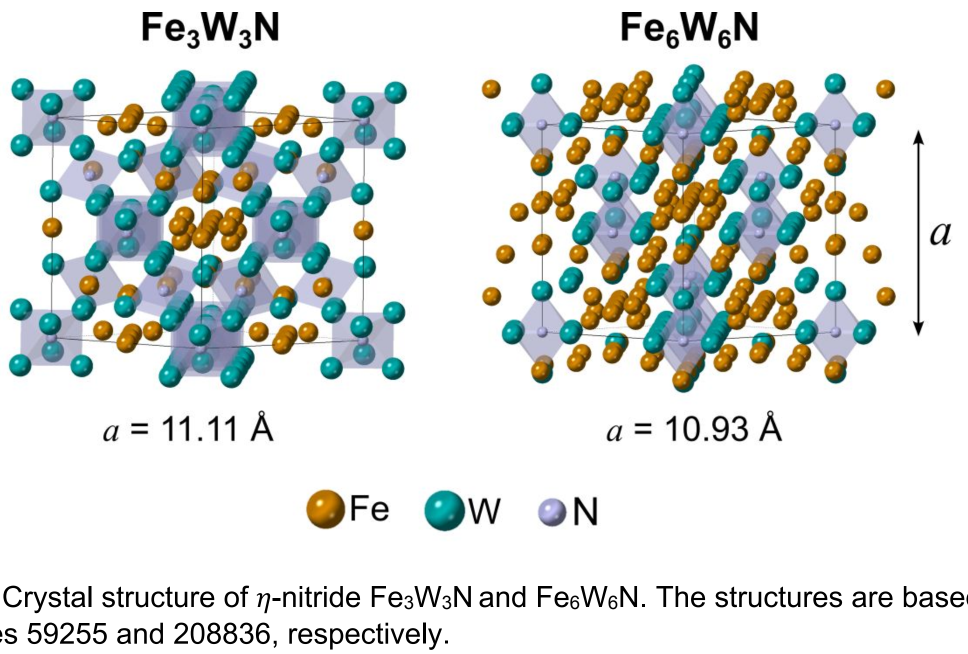

Figure S8. Crystal structure of η-nitride Fe_3W_3N and Fe_6W_6N. The structures are based on ICSD entries 59255 and 208836, respectively.

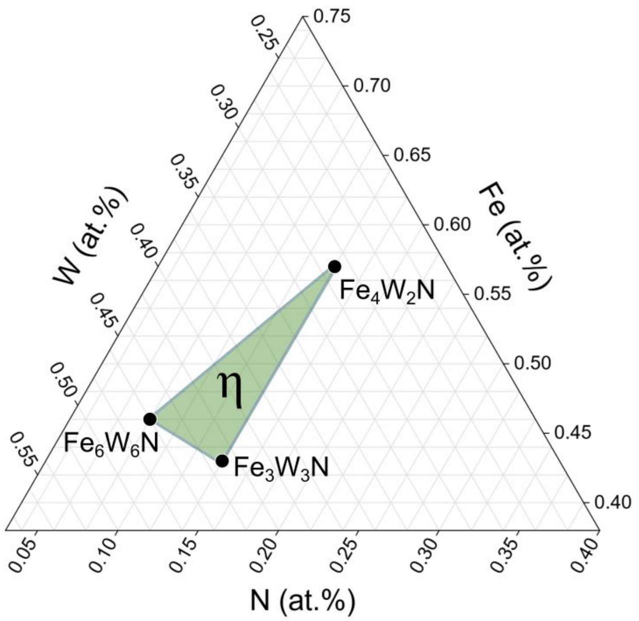

Figure S9. Ternary diagram Fe-W-N highlighting the tie line of compositions that exhibit the η crystal structure.

IV. Magnetic Measurements

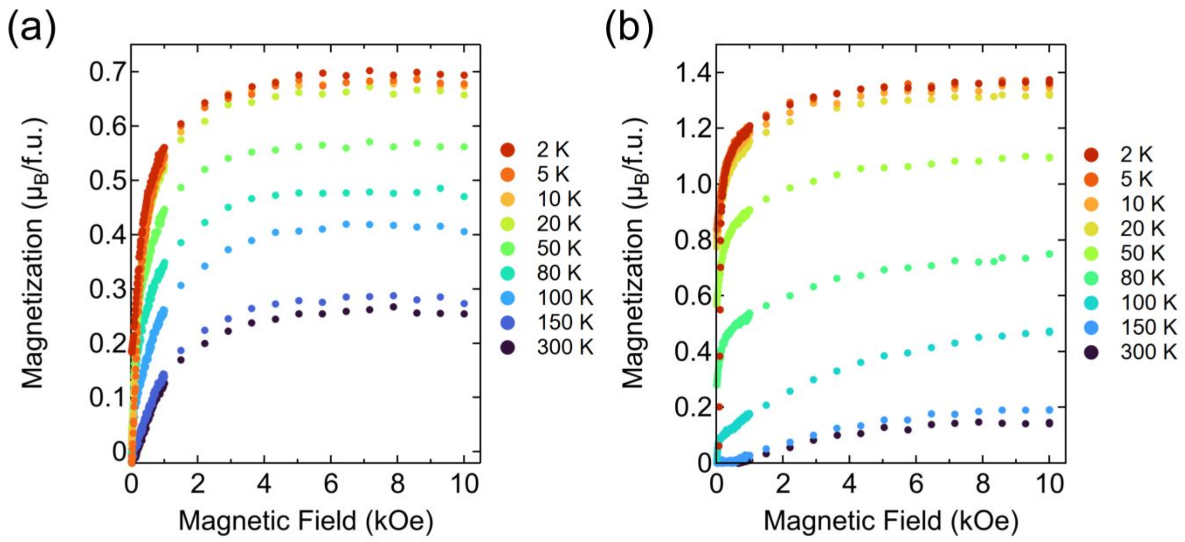

Figure S10. High-field magnetization measurements of Fe-rich films (a) Fe_3W_3N (x = 0.64) and (b) Fe_3Mo_3N (x = 0.59). In Fe_3W_3N, some additional slope correction at high field is applied due to some diamagnetic background bringing down the magnetization. In Fe_3Mo_3N, the magnetization does not seem to reach saturation at 10 kOe and keeps increasing linearly.

Additional details on methods for measuring and processing magnetic data of thin films

The magnetic properties of selected Fe-W-N and Fe-Mo-N thin films on Si/SiN_x substrate were measured in a Physical a Quantum Design DynaCool Physical Property Measurement System (PPMS) using the AC measurement system (ACMS) option which is based on vibration sample magnetometry (VSM) technique. We note that a “sample” here include film + substrate. Each sample was diced into ~ 5 x 5 mm pieces. Before loading in the instrument, the actual sample dimensions were measured as well as the mass. The thickness of the film was estimated by XRF. The sample was then mounted to a quartz measurement rod using insulating varnish (model VGE-7031) on the backside of the sample. The varnish was left on air to cure for about 30 min, and the sample was then loaded in the instrument. To remove the contribution of the substrate and obtain the film properties only, a piece of bare Si/SiN_x substrate around the same size was also measured in the same condition.

The magnetic moment of the film only μ_{film} is obtained by subtracting the magnetic moment of the substrate only $\mu_{substrate}$ (measured in units of emu) to the magnetic moment of the sample μ_{sample} (measured in units of emu). A scale factor, accounting for mass differences between the sample and the substrate pieces, is applied in the subtraction. The resulting moment (in units of emu) is expressed as:

$$\mu_{film} = \mu_{sample} - \frac{m_{sample}}{m_{substrate}} \mu_{substrate} \; (1)$$

The next step is to normalize the magnetization of the film by its mass and ultimately by formula units of Fe_3W_3N or Fe_3Mo_3N. Since it is not possible to measure the real mass of the film only, this conversion assumes that the film is fully composed of the material investigated (i.e. Fe_3W_3N or Fe_3Mo_3N) and does not take into account any impurities or secondary phases.

First, the volume of the film V_{film} is estimated using the area of the sample A_{sample} and the thickness of the film d_{film}:

$$V_{film} = A_{sample} \times d_{film} (2)$$

The mass of the film m_{film} is then estimated from the theoretical density ρ of the material investigated, assuming no other phases:

$$m_{film} = \rho \times V_{film} (3)$$

The mass magnetization of the film M_g (in emu/g) is obtained as:

$$M_g = \frac{\mu_{film}}{m_{film}} (4)$$

Finally, the magnetization per formula unit is expressed in term of Bohr magneton (μ_B/f.u.):

$$M = \frac{M_g}{9.274 \times 10^{-21}} \times \frac{M_{molar}}{N_A} (5)$$

The second term on the right represents the mass of one formula unit of material where M_{molar} is the molar mass of the material (in g/mol) and N_A the Avogadro number (in mol^{-1}).

The magnetization can then be expressed per Fe atom by simply dividing per number of Fe per formula unit, i.e. 3 for Fe_3W_3N or Fe_3Mo_3N.

In the case of magnetic susceptibility, the magnetic moment μ_{film} (in emu) is normalized by moles of formula unit of material and then divided by the applied field to obtain the susceptibility in unit of emu/mol-f.u.

V. Mixed Chemical Potential – Composition Phase Diagrams

Thin film nitride synthesis of Fe-M-N ternary nitrides, where M = Mo, W, possess thermodynamic boundary conditions that are open to the exchange of nitrogen with an external reservoir, and closed to the metallic composition ratio of Fe/(Fe+M). Thus, phase stability is evaluated using a mixed nitrogen chemical potential – metallic composition phase diagram. Each phase i, in the ternary Fe-M-N system is defined by the all-intensive energy potential ϕ_i which is a concave hyper-plane in $\phi - \mu_{Fe} - \mu_M - \mu_N$ space with composition coefficients of $N_{Fe,i}$, $N_{M,i}$, and $N_{N,i}$. SciPy's half space intersection algorithm [1] is then used to compute the

stability domain of each phase in $\mu_{Fe} - \mu_M - \mu_N$ space by computing the lower half-space envelop of all ϕ_i corresponding to all the phases.

Geometrically, the stability domains of single phases are 2-dimensional polygons in $\mu_{Fe} - \mu_M - \mu_N$ space, with the two-phase coexistence regions being 1-dimensional edges where two polygons meet, and three-phase coexistence regions being vertices where three polygons meet. For the $\mu_N - x_{Fe/(Fe+M)}$ diagrams, the vertical single-phase lines are plotted by extracting the projection of the single-phase polygons onto the μ_N axis. The 2-phase coexistence rectangles are plotted by identifying pairs of polytopes with shared vertices that form 2-dimensional edges, with each edge defining a minimum and maximum μ_N which defines the height of the rectangle, while the two compositions of the coexisting phases define the rectangle width. Finally, the horizontal three phase coexistence lines are plotted by identifying triplets of polygons with a single shared vertex, which defines the μ_N value at which the horizontal line appears, while the three compositions of the coexisting phases define its horizontal extent.

References:

[1] C. B. Barber, D. P. Dobkin, and H. Huhdanpaa, “The quickhull algorithm for convex hulls,” *ACM Trans Math Softw*, vol. 22, no. 4, pp. 469–483, Dec. 1996, doi: 10.1145/235815.235821.
